# Fatty acid amide hydrolase levels in brain linked with threat-related amygdala activation

**DOI:** 10.1016/j.ynirp.2022.100094

**Published:** 2022-06

**Authors:** Duncan GJ. Green, Duncan J. Westwood, Jinhee Kim, Laura M. Best, Stephen J. Kish, Rachel F. Tyndale, Tina McCluskey, Nancy J. Lobaugh, Isabelle Boileau

**Affiliations:** a Addiction Imaging Research Group, Canada; b Human Brain Lab, Canada; c Campbell Family Mental Health Research Institute, Canada; d Brain Health Imaging Centre, Centre for Addiction and Mental Health, 250 College Street, Toronto, ON, Canada; e Departments of Psychiatry, Canada; f Pharmacology & Toxicology, Canada; g Faculty of Medicine, University of Toronto, Toronto, Canada; h Department of Psychology, Korea University, Republic of Korea

**Keywords:** FAAH, fMRI BOLD, PET [^11^C]CURB, Threat, Perceptual face task, Amygdala

## Abstract

**Background:**

Preclinical evidence suggests that increasing levels of the major endocannabinoid anandamide decreases anxiety and fear responses potentially through its effects in the amygdala. Here we used neuroimaging to test the hypothesis that lower fatty acid amide hydrolase (FAAH), the main catabolic enzyme for anandamide, is associated with a blunted amygdala response to threat.

**Methods:**

Twenty-eight healthy participants completed a positron emission tomography (PET) scan with the radiotracer for FAAH, [^11^C]CURB, as well as a block-design functional magnetic resonance imaging session during which angry and fearful faces meant to activate the amygdala were presented.

**Results:**

[^11^C]CURB binding in the amygdala as well as in the medial prefrontal cortex, cingulate and hippocampus correlated positively with blood-oxygen-level-dependent (BOLD) signal during processing of angry and fearful faces (p_FWE_ < 0.05).

**Conclusion:**

Our finding that lower levels of FAAH in amygdala, medial prefrontal cortex, cingulate and hippocampus was associated with a dampened amygdala response to a threatening social cue aligns with preclinical and neuroimaging studies in humans and suggests the involvement of FAAH in modulating stress and anxiety in humans. The current neuroimaging study also lends support for the potential use of FAAH inhibitors to control amygdala hyperactivity, which is known to be involved in the pathophysiology of anxiety and trauma-related disorders.

## 1 Introduction

The amygdala plays a critical role in emotion processing and in the expression of adaptive responses to threat (i.e., fear)(LeDoux, 2014). Abnormal fronto-amygdala circuit function has been linked to vulnerability to various psychopathologies (e.g.: hypervigilance, anxiety) and to the development of psychiatric illnesses. In this regard, previous neuroimaging studies have shown a link between heightened amygdala activation following exposure to faces evoking anger and fear, and disorders such as posttraumatic stress (Shin et al., 2005) and anxiety disorders (Etkin et al., 2004), in which deficient responses to stress and extinguishing fear responses (i.e.: fear extinction learning) are characteristic (LeDoux, 2014). Impairments in fear extinction and maladaptive stress responses are core features of many complex neuropsychiatric disorders and thus are key targets for medication development.

Accumulating preclinical and clinical evidence suggests that the endocannabinoid system (ECS) and in particular the enzyme fatty acid amide hydrolase (FAAH) might play an important role in response to fear and stress and in the control of amygdala circuit (Gunduz-Cinar et al., 2013a), and as such has been the focus of medication development (Patel et al., 2017). FAAH is the primary catabolic enzyme for the major endocannabinoid N-arachidonoylethanolamine (anandamide, AEA), a lipid neurotransmitter for the cannabinoid type 1 receptor (CB1) that is densely expressed in the brain, including in the amygdala and medial prefrontal cortex (mPFC). The distribution of FAAH in brain overlaps with that of CB1 receptors and as such, it is functionally positioned to regulate ECS activity in brain regions that mediate stress and fear responses (Egertova et al., 2003).

In line with abundant preclinical literature demonstrating that decreased FAAH levels in basolateral amygdala of rodents, prevents and/or dampens behavioral, endocrine (i.e.: Hypothalamic-Pituitary Adrenal (HPA) axis activity) and physiological response to stress (Patel et al., 2017), recent studies in humans have provided preliminary evidence that pharmacological inhibition of FAAH reduces physiological and behavioral stress responses. FAAH inhibition enhances fear extinction in healthy controls and reduces feelings of anxiety in people with social anxiety disorders (Mayo et al., 2019; Schmidt et al., 2020). Echoing these findings, studies in people who have inherited lower FAAH activity through a Single Nucleotide Polymorphism (SNP) (rs324420, C385A, P129T) have shown that lower FAAH is associated with lower trait anxiety and responses to pain (Cajanus et al., 2016; Hariri et al., 2009a; Habib et al., 2019). Neuroimaging studies in humans using resting state functional and task-based magnetic resonance imaging (fMRI) have also shown that genetically decreased FAAH is associated with increased amygdala-mPFC connectivity as well as a blunted or desensitized amygdala response to threatening faces (Dincheva et al., 2015; Hariri et al., 2009b). Extending these imaging genetics studies, our group has recently reported a relationship between lower FAAH levels in brain, as measured directly by positron emission tomography (PET) imaging of the radiotracer for FAAH [^11^C]CURB, and stronger amygdala - mPFC and amygdala – anterior cingulate resting state connectivity (Green et al., 2021).

In the current study, we combined PET/[^11^C]CURB with task-related fMRI in healthy participants to examine whether amygdala [^11^C]CURB binding is related to amygdala response to threat. We also examine [^11^C]CURB binding in other areas that exert top-down inhibitory control over amygdala and that have been implicated in fear-learning and processing including the mPFC, cingulate (Feng et al., 2016) and hippocampus (Lisboa et al., 2010). Based on previous literature (Dincheva et al., 2015; G ä rtner et al., 2019), we hypothesized that higher [^11^C]CURB binding in our primary region of interest (ROI), the amygdala would be correlated with a greater amygdala response to angry and fearful faces.

## 2 Materials and methods

### 2.1 Subjects

Thirty healthy participants were recruited using online and community based advertisements. Participants provided written informed consent to complete a [^11^C]CURB PET and an MRI scan as part of two studies approved by the local Research Ethics Board. [^11^C]CURB PET and resting state fMRI data from all participants have been previously published (Green et al., 2021; Boileau et al., 2016; Boileau et al., 2015a). Participants with significant medical conditions, neurological illnesses, head trauma, Axis I psychiatric disorders as per Semi-structured Clinical Interview for Diagnostic and Statistical Manual of Mental Disorders (DSM-IV)(First et al., 1997) or MRI and PET contraindications (including pregnancy) were excluded. Urine on scan days (Rapid Response BTNX Inc., Markham, Canada) and scalp hair samples at screening (United States Drug Testing Laboratories, Des Plaines, IL, USA) were used to exclude participants with recent drug use. FAAH C385A rs324420 was genotyped according to published procedures as this affects [^11^C]CURB quantification (Boileau et al., 2015a). Urine samples were taken to rule out pregnancy and recent drug use. Recent alcohol and tobacco use, determined by breath-alcohol and expired carbon monoxide (>10 ppm) measurements respectively, were also assessed prior to the PET scan. Smokers were instructed not to smoke on the day of the scan.

### 2.2 PET image acquisition, reconstruction and processing

[^11^C]CURB radiosynthesis has been described previously (Wilson et al., 2011). PET was performed on a HRRT brain tomograph (CPS/Siemens, Knoxville, TN, USA) as described in Rusjan et al., 2013) (Rusjan et al., 2013). Briefly, participants received an injection of 342 ± 33 MBq of [^11^C]CURB while supine with their head secured by a thermoplastic mask. Emission data were then acquired for 1 h in sequential frames of increasing duration (Boileau et al., 2015a). Images were reconstructed from 2D sinograms with a 2D filtered-back projection algorithm, using a HANN filter at Nyquist cutoff frequency. Radioactivity in arterial blood was counted with an automatic blood sampling system (Model PBS-101, Veenstra Instruments, The Netherlands) for the first 22.5 min after injection. The radioactivity in plasma and the metabolism was measured from arterial blood samples extracted at 3, 7, 12, 20, 30, 45 and 60 min after injection. A metabolite-corrected plasma curve was generated and used as the input function for the kinetic analysis (Rusjan et al., 2013). Blood-to-plasma radioactivity ratios were interpolated by a biexponential function and the parent plasma fraction was interpolated by a Hill function.

Time-activity curves in the bilateral amygdala and from whole brain ROIs (including striatum, hippocampus, mPFC, and the cingulate, occipital, inferior parietal, and temporal cortices) were extracted using ROMI as described in (Rusjan et al., 2006). Regional time activity curves were analyzed using a 2-TCMi as described in (Rusjan et al., 2013) generating regional λk_3_ (λk_**3**_ = k_3_*k_1_/k_2_), the outcome measure for FAAH levels. Amygdala λk_3_ was the variable of interest in the current study.

### 2.3 MRI data acquisition

MRI data were acquired using a 3-T GE Discovery MR750 with an 8-channel radiofrequency head coil (General Electric, Milwaukee, WI, USA). Proton-density (PD) images were obtained for PET co-registration and region of interest (ROI) delineation (spin echo imaging, TR = Min/Full, ∼6s, TE = 13.7 ms, ETL = 8, receiver BW ± 15.6 kHz, FOV = 220 mm × 220 mm, matrix = 256 × 256, slice thickness = 2 mm). Sagittal T1-weighted structural MRIs were obtained for fMRI co-registration and ROI delineation (TR = 6.7 ms, TE = 3 ms, TI = 600 ms, flip angle = 8°, field of view = 230 × 230 mm, matrix size = 256 × 256 × 200, voxel size = 0.9 mm (Etkin et al., 2004)). fMRI data were obtained over one run of 8 min and 53 s using a T2*-weighted spiral in/out 2D gradient echo sequence (Glover and Law, 2001) (TR = 2500 ms, TE = 30 ms, flip angle = 70°, slice thickness = 3.0 mm, 39 sequential slices with no gap, field of view = 200 × 200 mm, matrix size = 64 × 64, voxel size = 3.1 × 3.1 × 3.0 mm). A total of 168 vol were obtained per scan.

### 2.4 fMRI amygdala reactivity paradigm

Participants performed a single-run facial emotion matching-task adapted from Hariri et al. (2009) using angry and fearful faces from the Ekman and Friesen face set (Ekman and Friesen, 1976). During the task they responded to two types of conditions: in one, they were instructed to match a target face to one of three emotional faces displayed below and in the other they were asked to match shapes to a target shape. The task was run using a single run block-design, which used an ABAB interleaved pattern. There were 4 test blocks and 5 control blocks, lasting a total of 8 m 53s. Each 48s test block had 6 trials and had a variable inter-stimulus interval of 2–6s Each 36s. control block had 6 trials and had a fixed inter-stimulus interval of 2s. Instructions were displayed for 2s before each block in both test and control conditions. Stimuli in both the control and test condition were displayed for 4s.

### 2.5 fMRI data preprocessing and analysis

Analyses were conducted using FMRIB Software Library (FSL, http://www.fmrib.ox.ac.uk/fsl) and Statistical Parametric Mapping 12 (SPM12, Wellcome Trust Centre for Neuroimaging, London, UK, www.fil.ion.ucl.ac.uk/spm). The first four volumes of each scan were discarded for magnetization equilibrium, leaving 164 vol for analysis. MRI sequence-specific artifacts were removed using FSL's ICA-based denoising tool (MELODIC, www.fmrib.ox.ac.uk/fsl/melodic/html) separately on the spiral-in and spiral-out images. Artefact-related components were removed using FSL's FIX toolbox (FIX toolbox, http://fsl.fmrib.ox.ac.uk/fsl/fslwiki/FIX)(Salimi-Khorshidi et al., 2014). This procedure included automated removal of non-brain tissue (BET (Smith, 2002)) and head-motion correction (MCFLIRT (Jenkinson et al., 2002)). The cleaned spiral-in and spiral-out images were combined with a weighted average (Glover and Law, 2001) and registered to the structural image using FLIRT. To correct for motion, images for each participant were realigned to the first retained volume in the series. The functional data were normalized using affine transformation to the SPM EPI template in standard space followed by nonlinear registration to the EPI template and resampling to 2 mm isotropic voxels using 4th order spline interpolation (Calhoun et al., 2017). The normalized fMRI data were then spatially smoothed with a 6 mm FWHM Gaussian kernel.

We used linear contrasts employing canonical hemodynamic response functions to calculate Blood Oxygenation Level Dependent (BOLD) activation for each condition. Six head movement parameters were added as a regressor into the General Linear Model (GLM) to account for the confounding effect of movement.

In order to reveal brain regions involved in emotional face-specific processing, we generated the activation map for the face condition contrasted with the shape condition [contrast = 1, −1] at face > shape per subject. The amygdala activation differences were extracted from these individual contrast images were entered to the further analyses (below).

In the group level analysis, we first performed a one sample *t*-test on the individual contrast image (face > shape condition) to test amygdala involvement during emotional face processing. Both fear and angry faces were pooled to increase power and because previous studies have failed to find different amygdala responses to these stimuli (Mattavelli et al., 2014). We opted to compare faces with shapes as this contrast provides the most robust activation of the amygdala compared to neutral faces which have been shown to elicit an amygdala response (Rauch et al., 2003). Based on our *a priori* hypothesis and specific research interest in the amygdala, the significance for all group-level analyses was determined using a small volume correction Family-Wise Error Rate (FWE)-corrected threshold of p < 0.05. The bilateral anatomical amygdala ROI was constructed using the WFU Pickatlas (Maldjian et al., 2003). Visual inspection was conducted for all participants to ensure that the anatomical amygdala mask covered the amygdala for each subject. All coordinates presented going forward are presented using the MNI coordinate system.

Next, we examined whether amygdala FAAH level is related to amygdala activation (% BOLD change) during angry/fearful face processing. [^11^C]CURB λk3 values were extracted from the bilateral amygdala for each subject. These values were then used as a regressor in a voxel-wise correlation with the face > shape contrast images, similar to what was used in Hariri et al. (2009). Significance for analysis was small volume correction FWE at p < 0.05.

To examine confounding effects on this relationship, we extracted the mean cluster level percent signal change for the significant amygdala voxels the face > shape contrast conditions using MarsBaR (Brett et al.,) and analyzed using SPSS. Additional analyses of the effect of trait neuroticism were constrained to voxels that were found to be significantly correlated with amygdala [^11^C]CURB λk_3_. Covariate analysis (including smoking status, age, sex, ethnicity, and body mass index (BMI)) were conducted using partial correlations in order to determine any confounding effects. The covariates were chosen based on the reported effects of age (Gee et al., 2016; Maccarrone et al., 2002) sex (Riebe et al., 2010), BMI (Tour ĩ o et al., 2010), nicotine (Cippitelli et al., 2011) and FAAH C385A SNP on FAAH activity. In order to examine the regional extent of a finding, we contrasted the mean percent signal change for the significant amygdala voxels in the face > shape contrast conditions with [^11^C]CURB binding from other ROIs including the mPFC, cingulate, and hippocampus involved in fear-learning and processing (Feng et al., 2016; Lisboa et al., 2010) as well as other brain regions including the ventral striatum, temporal, occipital, and parietal cortices used to assess generalizability/specificity of the finding.

## 3 Results

### 3.1 Demographics

In total, 30 participants completed the study. One participant was excluded for excessive spiral artefact and one participant was excluded for excessive movement (>2 mm). 28 participants’ data were included in the final analyses (16 female). Demographic information is outlined in Table 1. [^11^C]CURB PET data from all participants have been previously published (Green et al., 2021; Boileau et al., 2015a; Boileau et al., 2015b).

**Table 1:** Demographics of our sample.

# of subjects	28
Sex (M, F), n	12, 16
Age (mean ± SD)	30.41 (11.77)
Ethnicity (White/Asian/Black)	16/4/8
Body Mass Index (mean ± SD)	24.16 (2.85)
FAAH Genotype rs324420 C385A (C/C, A/C, A/A), n	18, 9,1
Education, Years (mean ± SD)	15.71 (2.37)
Daily Cigarette Smokers, n	6
Cigarettes/Day	13.83 (range: 3–25)
Fagerstrom Test for Nicotine Dependence	4.3 (range: 1–8)
Amount Injected (mCi) (mean ± SD)	9.56 (0.77)
Specific Activity (mCi/μmol) (mean ± SD)	2703.36 (1078.69)
Mass Injected (μg) (mean ± SD)	1.27 (0.48)

### 3.2 Amygdala BOLD reactivity

**Fig. 1:**
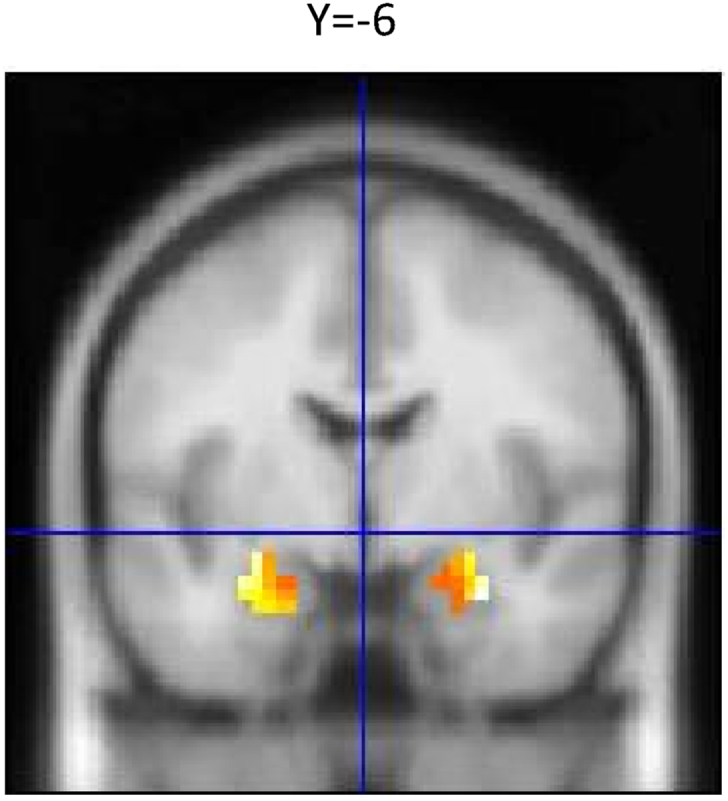
**Amygdala Activation to emotional faces compared to shapes.** Amygdala BOLD reactivity was increased in response to emotional faces vs shapes (R, 30, −4, −23, k = 30; L, −27, −7, −20, k = 35, All p _FWE small volume corrected_ < 0.05).

Voxel-wise one-sample t-tests were conducted in order to ensure proper activation of the amygdala in response to angry/fearful faces. We found that angry/fearful faces reliably increased amygdala activity compared to geometric shapes in both the left (x, y, z: −27, −7, −20, k = 35, p_FWE_ _small volume corrected_ < 0.05) and right amygdala (x, y, z: 30, −4, −23, k = 30, p_FWE small volume corrected_ < 0.05; (Fig. 1). We found no correlation between amygdala response to angry/fearful faces and age and no sex differences (p > 0.2).

### 3.3 Amygdala [^11^C]CURB binding and amygdala BOLD reactivity

**Fig. 2:**
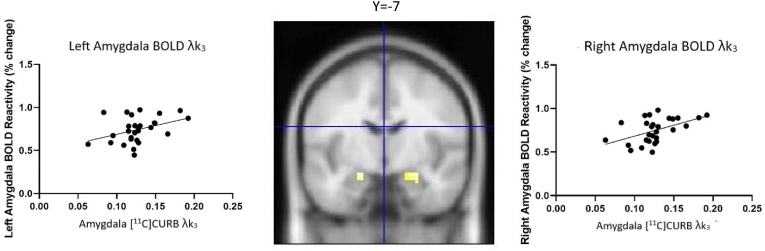
**Correlation between amygdala BOLD reactivity and amygdala [**^**11**^**C]CURB.** Amygdala BOLD reactivity in response to emotional faces was positively correlated with [^11^C]CURB binding (R, 27, −7, −16, k = 17, r = 0.51 p_FWE small volume corrected_ < 0.01, z = 2.43; L, −18, −7, −16, k = 8, r = 0.39, p_FWE small volume corrected_ = 0.03, z = 1.74).

The voxel-wise correlation analysis between [^11^C]CURB λk_3_ in the amygdala and amygdala ROI BOLD signaling during angry/fearful face processing revealed a significant positive correlation (right amygdala (x, y, z), 27, −7, −16, k = 17, r = 0.51 p_FWE small volume corrected_ < 0.01, z = 2.43; left amygdala (x, y, z), −18, −7, −16, k = 8, r = 0.39, p_FWE small volume corrected_ = 0.03, z = 1.74) (Fig. 2). When controlling for age, sex, smoking status, BMI, and ethnicity, the correlation between amygdala [^11^C]CURB λk_3_ and amygdala BOLD maintained significance (left amygdala (x, y, z) −18, −7, −16, k = 8 p = 0.03, r = 0.40) (right amygdala (x, y, z) 27, −7, −16, k = 17, p = 0.01, r = 0.47).

We examined the effect of [^11^C]CURB λk_3_ from other ROIs on the mean amygdala BOLD activation cluster from the principle reported findings. We found that [^11^C]CURB λk3 from the hippocampus were significantly correlated with right and left amygdala activation whereas [^11^C]CURB λk3 from the hippocampus as well as the bilateral mPFC and cingulate were significantly correlated with the right amygdala. No correlations with other ROIs were significant (see Supplementary Table 1). We tested whether the FAAH C385A genotype affected the BOLD reactivity in response to angry/fearful faces in the amygdala using a two-sample *t*-test. We did not find an effect of C385A genotype on amygdala BOLD reactivity (right amygdala p = 0.41, left amygdala p = 0.25) (data not shown). It is possible that we were underpowered to find significant genotype effects. There were no significant sex differences in outcome relationships.

## 4 Discussion

We found that levels of the ECS enzyme FAAH measured *in vivo* in human brain as inferred from [^11^C]CURB binding are positively associated with bilateral amygdala response to threat. Our findings support our hypothesis and provide further evidence for earlier neuroimaging studies in humans that have linked the minor FAAH C385A gene variant with lower stress-reactivity (Mayo et al., 2018) and response to threat (Hariri et al., 2009b) and greater habituation to repeated exposure to threat (Gunduz-Cinar et al., 2013b). Together these findings suggest that (state and/or trait) variability in brain levels of FAAH may contribute to inter-individual differences in neural processes that mediate stress-related behaviors and that decreased hydrolytic breakdown of AEA in brain might dampen neural response to threatening stimuli.

Our findings provide support to recent, preliminary findings from a clinical trial in social anxiety disorder, which suggests that a 12-week daily regimen of the FAAH inhibitor JNJ-42165279 nominally improves scores on the Liebowitz Social Anxiety Scale as well as the Clinical Global Impression-Improvement scale (Schmidt et al., 2020). In line with this finding and with the notion that FAAH inhibitors could buffer response to fear, a 10-day sub-chronic regimen of the FAAH inhibitor PF-04457845 has been shown to potentiate fear extinction and produced decreased physiological and affective responses to a stressful stimulus in healthy controls (Schmidt et al., 2020). Together these clinical findings provide evidence of the mechanism of action for the use of non-psychogenic FAAH inhibitors in disorders involving poor control of fear response such as post-traumatic stress for which the potential use of cannabis has already been proposed (Mayo et al., 2021). Further clinical evidence shows that administration of FAAH inhibitors decreases amygdala BOLD reactivity to threatening stimuli in healthy males, indicating a potential marker for decreased stress and anxiety (Paulus et al., 2020).

Animal models investigating fear extinction, a behavioral assay for translational studies of posttraumatic stress and anxiety disorders, have suggested that infusion of AEA (Munguba et al., 2011), FAAH inhibitors or AEA reuptake blockers (Mayo et al., 2018) in the basolateral amygdala (with high levels of CB1 receptors and of FAAH) as well as deletion of FAAH gene (Wise et al., 2009) can facilitate fear extinction. Inversely, exposure to acute and chronic stress (for example, restraint stress, foot shock) rapidly upregulates FAAH in the basolateral amygdala (Gray et al., 2015; Hill et al., 2009, 2013). Experimental animal models have further demonstrated that modulation of extinction of conditioned fear responses is through an action on CB1 receptors in the amygdala but also involves mPFC neurons which receive afferents from amygdala (Conde et al., 1995). In the current study we found that FAAH levels in three regions outside of the amygdala (mPFC, hippocampus and cingulate) also correlated positively with BOLD response to threatening faces. Further reference regions, the ventral striatum and temporal, parietal, and occipital lobes, did not correlate significantly with BOLD response to threatening faces. As our current imaging methods do not allow us to distinguish distinct nuclei it is unclear whether reduced FAAH-related fear responses are through the (whole) amygdala, through some of its functionally distinct nuclei (i.e.: basolateral vs lateral as suggested by some animal studies) or to what extent this relationship involves other nodes of the fear circuit (e.g.: vmPFC, cingulate, hippocampus). Similarly, although the AEA/CB1 receptor interaction is likely involved in the effects on BOLD, other receptor systems and FAAH substrates could be involved. For example, palmitoylethanolamide (PEA), N-oleoylethanolamide (OEA) docosahexaenoylethanolamide (DHEA) are non-endocannabinoid substrates for FAAH. Lower FAAH would increase brain levels of these N-acylethanolamines which in some cases have been linked with response to stress (Dlugos et al., 2012) and could affect neural response to threat through the peroxisome proliferator-activated receptor-alpha (PPAR-α) or through vanilloid receptor 1.

The mechanism by which FAAH modulates amygdala response to fear is not entirely understood. Electrophysiological studies investigating the effects of increased AEA (through pharmacological or FAAH gene knock-out) on amygdala synaptic potentials suggest that increased AEA/CB1 signaling enhances long-term depression of inhibitory transmission (LTDi) leading to an activity-dependent suppression of GABA release from cholecystokinin (CCK)-positive GABAergic interneurons (enriched with CB1 receptors). The resulting effect would be a decrease in the activity of inhibitory interneurons in the amygdala, which would in turn enhance excitatory synaptic transmission within main output sites in the amygdala and facilitate top-down inhibitory control from vmPFC (Rabinak et al., 2014). In this context reduced FAAH may specifically engage neural circuits that exert control over amygdala (e.g.: vmPFC) and therefore in part control fear extinction learning.

Top-down inhibitory control from frontal regions, specifically the mPFC plays a critical role in fear extinction and emotion regulation in which CB1 dependent amygdala plasticity is believed to be necessary (Li et al., 2018). In this regard we have recently published findings consistent with earlier literature (Dincheva et al., 2015; G ä rtner et al., 2019), showing that lower FAAH levels in brain correlate with greater resting-state connectivity between amygdala and mPFC (Green et al., 2021). Interestingly we found that the strength of resting-state amygdala-mPFC connectivity reported in our previous study was correlated with amygdala BOLD response to threat. Connectivity measures from the earlier study were compared to the BOLD responses in the current study using the same cohort (n = 28: left amygdala: p = 0.01, r = −0.476; right amygdala: p = < 0.01, r = −0.522) (data not shown). Collectively our fMRI studies suggest that individuals with lower brain levels of FAAH (specifically in amygdala) have stronger amygdala-mPFC connectivity and lower amygdala reactivity to threat; in other words, lower response to threat may be in part explained by stronger connectivity in amygdala-mPFC circuit. This interaction between responsivity and connectivity reinforces the idea that a FAAH inhibitor would be an effective treatment for aberrant fear response in PTSD by increasing the top-down inhibitory control of frontal regions.

While our findings suggest a bilateral effect, there are mixed findings in the literature as to the laterality of fear processing where right (Sergerie et al., 2008; Baker and Kim, 2004) left (Hardee et al., 2008) and bilateral (Spohrs et al., 2018) amygdala responses have been reported. Similarly, other literature suggests a lateralized ECS response to threat (Roche et al., 2007).

Our data should be interpreted in light of the following limitations. Our study was done in a small sample of healthy participants limiting our ability to probe clinically meaningful bio-behavioral relationships. Our design pooling fear and angry faces and contrasting them to geometric shapes (sensorimotor control) instead of neutral faces does not allow dissecting the perceptual response to faces from the emotional responses per se. Our study was underpowered to replicate findings from earlier genotype studies (Hariri et al., 2009b). Further, a recent meta-analysis investigating test-retest reliability of common fMRI tasks, has indicated that the test-retest reliability of the perceptual face task may be too low to use as an appropriate biomarker, therefore caution is warranted when interpreting results (Elliott et al., 2020).

In conclusion, our exploratory findings that FAAH levels in brain, as measured through molecular imaging, correlate positively with BOLD response to threat in humans support the involvement of brain FAAH in fear-related disorders known to affect amygdala function and that FAAH inhibition could alter neural responses to threat.

## Conflict of interest declaration

Rachel F Tyndale has consulted for Quinn Emanuel and Ethismos on unrelated topics. Duncan GJ Green, Duncan J Westwood, Jinhee Kim, Laura M Best, Stephen J Kish, Tina McCluskey, Nancy Lobaugh, and Isabelle Boileau declare no conflict of interest.

This study was supported in part by The 10.13039/100000026National Institutes of Health and National Institute on Drug Abuse (NIH/NIDA R21 DA036024 (IB)), 10.13039/501100000024CIHR FDN-154294 (RFT), and a Canada Research Chair in Pharmacogenomics (RFT).
