# Supplementary material for: Fatty acid amide hydrolase levels in brain linked with threat-related amygdala activation
Source: Neuroimage Rep. Author manuscript; Available in PMC 2023 Jun 22. (PMC10206405; doi:10.1016/j.ynirp.2022.100094)
Supplement: 1 [file NIHMS1895392-supplement-1.docx]

**Supplemental Materials**

**Supplementary Table 1.** Correlations between left and right amygdala BOLD activation principle finding cluster and [^11^C]CURB λk_3_ in amygdala, medial prefrontal cortex (mPFC), cingulate, temporal cortex, inferior parietal cortex, occipital cortex, hippocampus, and ventral striatum.

|  | Amygdala*  [^11^C] CURB λk_3_ | mPFC  [^11^C] CURB λk_3_ | Cingulate Cortex  [^11^C] CURB λk_3_ | Ventral Striatum  [^11^C] CURB λk_3_ | Inferior Parietal Cortex  [^11^C] CURB λk_3_ | Occipital Cortex  [^11^C] CURB λk_3_ | Temporal Cortex  [^11^C] CURB λk_3_ | Hippocampus  [^11^C] CURB λk_3_ |
| --- | --- | --- | --- | --- | --- | --- | --- | --- |
| Amygdala  [^11^C] CURB λk_3_ |  |  |  |  |  |  |  |  |
| P | N/A | <0.01 | <0.01 | <0.01 | <0.01 | <0.01 | <0.01 | <0.01 |
| R | N/A | 0.91 | 0.84 | 0.86 | 0.82 | 0.81 | 0.81 | 0.87 |
| Right Amygdala BOLD cluster |  |  |  |  |  |  |  |  |
| P | <0.01 | 0.02 | 0.04 | 0.41 | 0.36 | 0.28 | 0.20 | 0.01 |
| R | 0.51 | 0.43 | 0.38 | 0.15 | 0.18 | 0.21 | 0.25 | 0.46 |
| Left Amygdala BOLD cluster |  |  |  |  |  |  |  |  |
| P | 0.03 | 0.10 | 0.13 | 0.24 | 0.42 | 0.33 | 0.24 | 0.02 |
| R | 0.39 | 0.35 | 0.27 | 0.22 | 0.16 | 0.19 | 0.23 | 0.44 |
